# Supplementary material for: Different ecological demands shape differences in population structure and behaviour among the two generations of the small pearl-bordered fritillary
Source: PeerJ. 2024 Feb 26;12:e16965. doi: 10.7717/peerj.16965 (PMC10903349; doi:10.7717/peerj.16965)
Supplement: Supplemental Information 1 [file peerj-12-16965-s001.pdf]

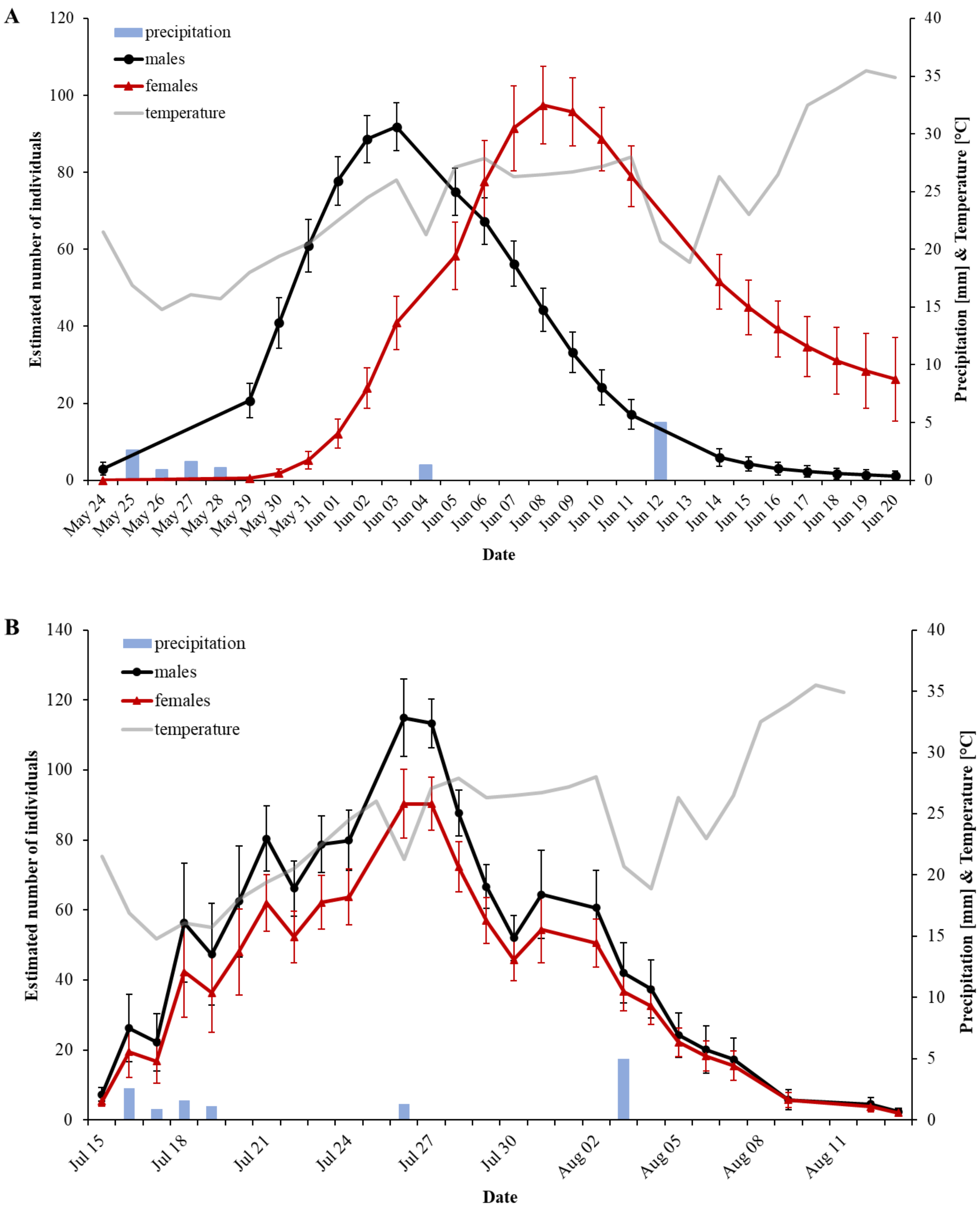

Figure S1 Estimated daily population sizes of the first (a) and second (b) generation of *Boloria selene* for females (red) and males (blue); as grey bars the daily precipitation amount in mm and as yellow line the daily maximum temperature in °C (scales on the right).
