# Supplementary material for: Different ecological demands shape differences in population structure and behaviour among the two generations of the small pearl-bordered fritillary
Source: PeerJ. 2024 Feb 26;12:e16965. doi: 10.7717/peerj.16965 (PMC10903349; doi:10.7717/peerj.16965)
Supplement: Supplemental Information 2 [file peerj-12-16965-s002.pdf]

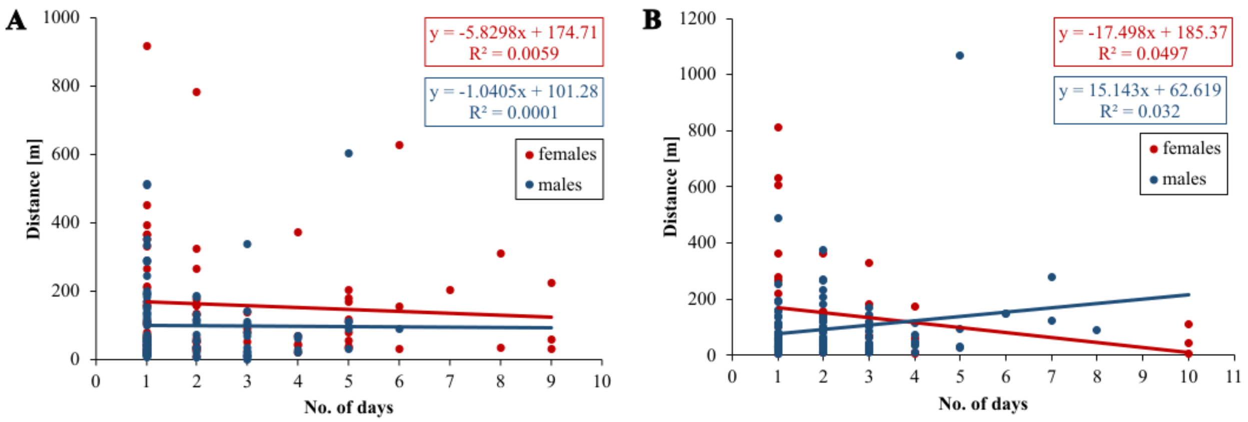

Figure S2 Correlation of flight distance between capture and first recapture of *Boloria selene* and the number of days in-between; males in blue and females in red in the first (a) and second (b) generation.
