# Supplementary material for: Different ecological demands shape differences in population structure and behaviour among the two generations of the small pearl-bordered fritillary
Source: PeerJ. 2024 Feb 26;12:e16965. doi: 10.7717/peerj.16965 (PMC10903349; doi:10.7717/peerj.16965)
Supplement: Supplemental Information 3 [file peerj-12-16965-s003.pdf]

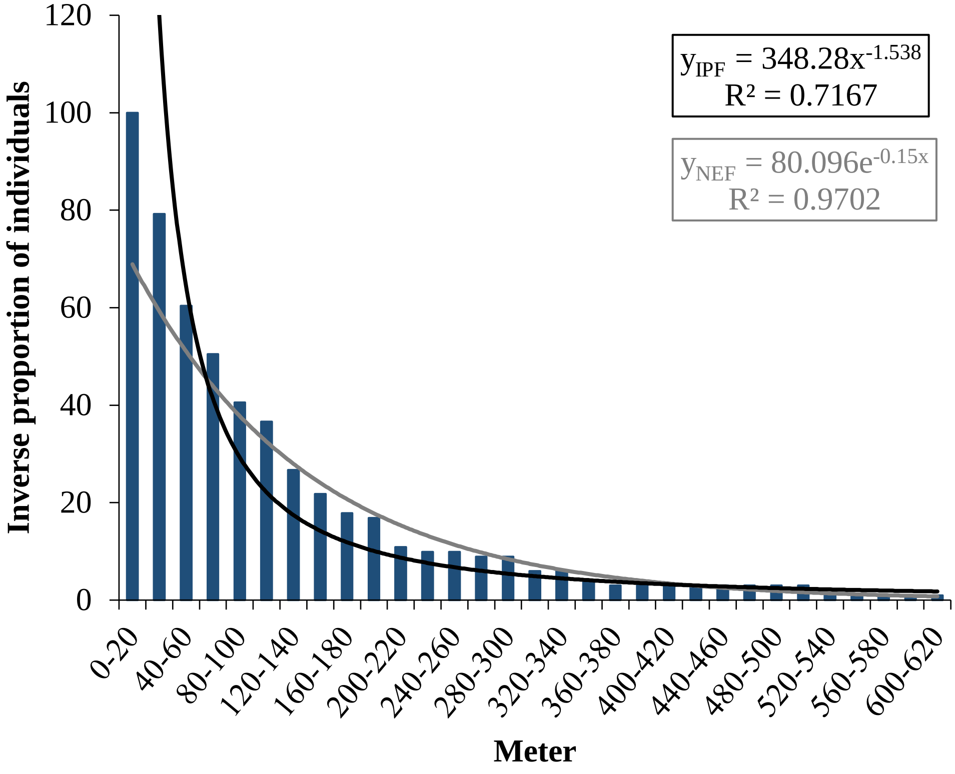

Figure S3 Inverse proportion of *Boloria selene* males in the first generation reaching certain 20 m distance classes; fitted NEF (grey line) and IPF (black line).
