# Supplementary material for: Different ecological demands shape differences in population structure and behaviour among the two generations of the small pearl-bordered fritillary
Source: PeerJ. 2024 Feb 26;12:e16965. doi: 10.7717/peerj.16965 (PMC10903349; doi:10.7717/peerj.16965)
Supplement: Supplemental Information 4 [file peerj-12-16965-s004.pdf]

A

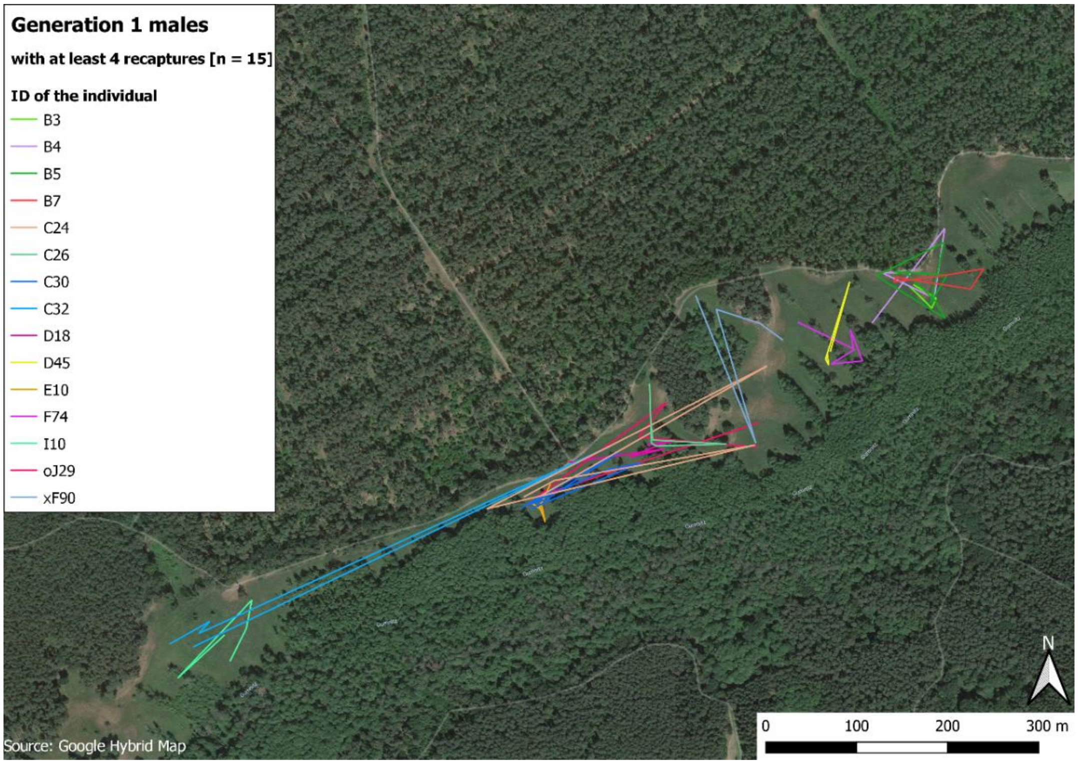

B

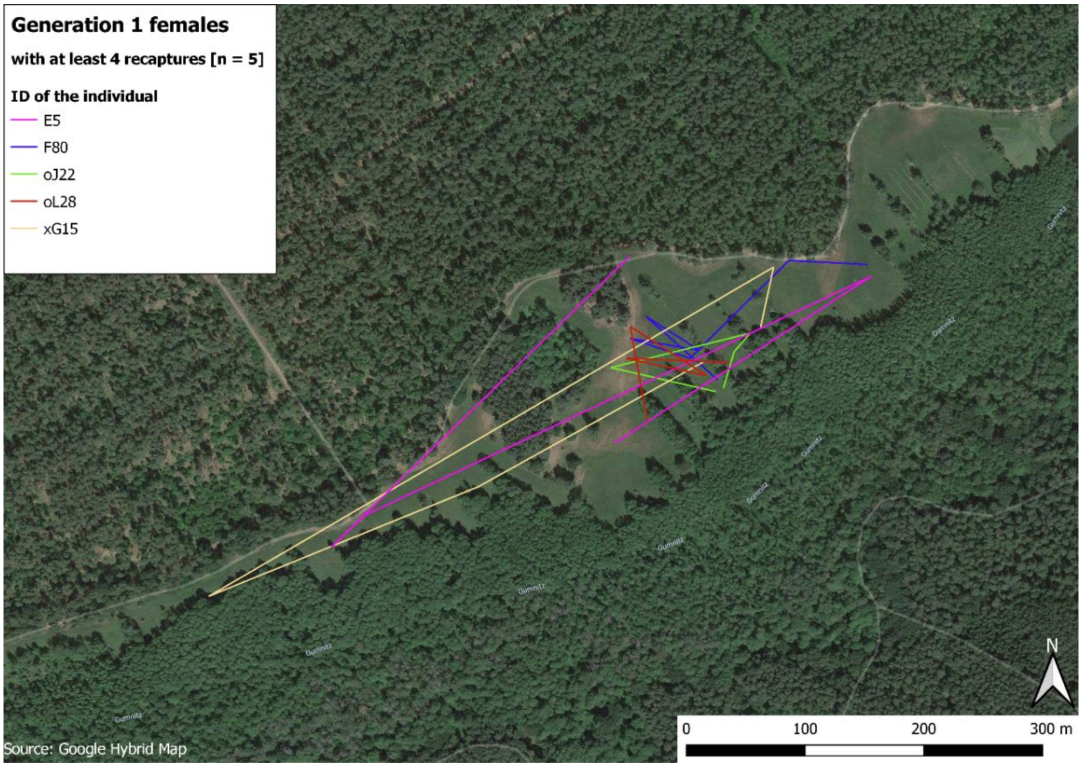

Figure S4 Flight paths of *Boloria selene* males (a) and females (b) in the first generation; for presentation, the minimum number of recaptures was set to 4, to show approximately equal numbers of flight paths.
