# Supplementary material for: Different ecological demands shape differences in population structure and behaviour among the two generations of the small pearl-bordered fritillary
Source: PeerJ. 2024 Feb 26;12:e16965. doi: 10.7717/peerj.16965 (PMC10903349; doi:10.7717/peerj.16965)
Supplement: Supplemental Information 6 [file peerj-12-16965-s006.pdf]

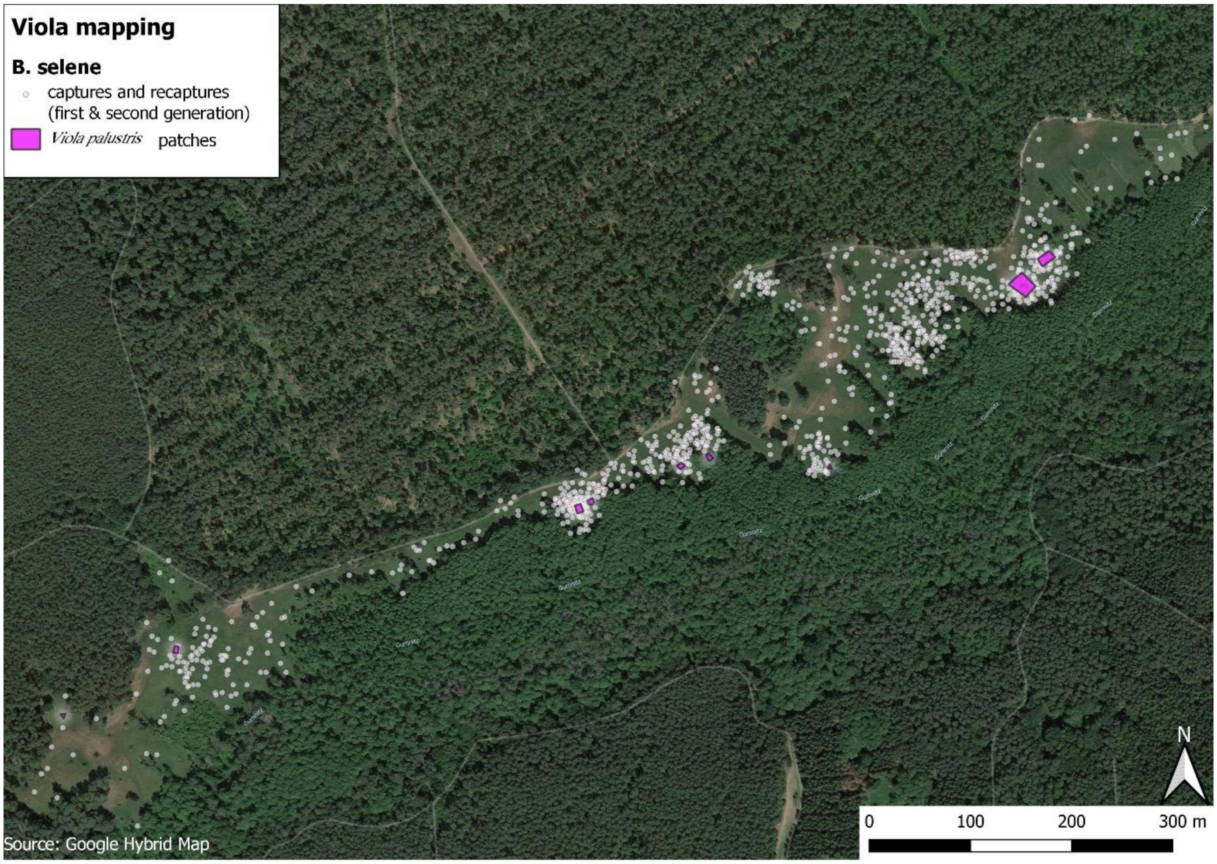

Figure S6 Visualisation of mapped *Viola palustris* patches as violet polygons; captures and recaptures of *Boloria selene* individuals in both generations as grey points.
