# Supplementary material for: Different ecological demands shape differences in population structure and behaviour among the two generations of the small pearl-bordered fritillary
Source: PeerJ. 2024 Feb 26;12:e16965. doi: 10.7717/peerj.16965 (PMC10903349; doi:10.7717/peerj.16965)
Supplement: Supplemental Information 7 [file peerj-12-16965-s007.docx]

**Table S1** Distances and number of days of lifespan movements for *Boloria selene* individuals in the first and second generation.

| **Generation 1** | **males** | **females** | **total** |
| --- | --- | --- | --- |
| Max. distance (m) | 1585 | 1723 |  |
| Mean distance (m) | 225.9 | 306.8 | 259.3 |
| Max. no. of days | 11 | 13 |  |
| Mean no. of days | 4.2 | 5 | 4.5 |
| **Generation 2** | **males** | **females** | **total** |
| Max. distance (m) | 1070 | 1158 |  |
| Mean distance (m) | 140.4 | 243.4 | 169.3 |
| Max. no. of days | 10 | 13 |  |
| Mean no. of days | 3.3 | 4.2 | 3.6 |
